# The impact of sulfonylureas on diverse ion channels: an alternative explanation for the antidiabetic actions

**DOI:** 10.3389/fcell.2025.1528369

**Published:** 2025-06-23

**Authors:** Xian-Tao Li, Meng-Ze Yun

**Affiliations:** School of Medicine, Jingchu University of Technology, Jingmen, China

**Keywords:** sulfonylureas, ion channels, alternative explanation, antidiabetic actions, side effects

## Abstract

The oral hypoglycemic drug sulfonylureas exhibit substantial therapeutic benefits for millions of patients with type 2 diabetes mellitus (T2DM), although with common adverse effects, such as hypoglycemia. It is generally believed that inhibition of K_ATP_ channels by sulfonylureas in pancreatic β-cells enables the insulin release to reduce glycemic levels, a primary mechanism underlying pharmacological effectiveness. Accumulated evidence reveals that multiple ion channels, such as Kv and TRP, are also expressed in β-cells in the pancreatic islets of Langerhans, and these channels, particularly Kv2.1, show important functional roles in tuning the electrical activity of β-cells, accordingly participating in the modulation of insulin secretion. Existing data reveal that several ion channels besides K_ATP_ channels could be directly blocked by sulfonylureas, and consequent membrane depolarization serves to facilitate the insulin release, possibly contributing to glycemic control or side effects. Furthermore, the modulation of sulfonylurea-mediated activation of Epac2A on diverse ion channels could produce the pharmacological efficacy, indicative of an indirect regulatory way. The scenario of sulfonylureas impacting diverse ion channels may provide an alternative explanation for the antidiabetic actions and side effects, extending our understanding of these classical clinic drugs.

## 1 Introduction

Sulfonylureas are ubiquitously employed as antidiabetic drugs for type 2 diabetes mellitus (T2DM) with characteristics of the deficiency of insulin secretion and/or insulin resistance (IR), a metabolic illness afflicting millions of people worldwide (Rorsman and Ashcroft, 2018). Generally, such drugs are capable of dampening the ATP-sensitive potassium channels (K_ATP_), which are abundantly expressed in β-pancreatic cells with an octameric structure composed of four inward rectifier Kir6.2 subunits and four regulatory SUR1 subunits (Inagaki et al., 1997). Sulfonylureas can depolarize the membrane potential to activate the voltage-dependent Ca^2+^ channels (VDCC), in turn causing influx of extracellular Ca^2+^ to stimulate insulin release (Inagaki et al., 1995), which is a primary mechanism underlying drug’s effects on glycemic control. Administration of these agents could generate diverse adverse effects, such as weight gain and hypoglycemia. The latter is the most common side effect, which can arise from all sulfonylureas, especially for long-acting sulfonylureas, including glibenclamide and chlorpropamide (Gangji et al., 2007). Nowadays, second generation sulfonylureas, such as glibenclamide, glimepiride, gliclazide and glipizide, are taken as oral hypoglycemic drugs in the clinic, with higher safety compared with first generation agents, such as tolbutamide and chlorpropamide (Sola et al., 2015). As a second-generation sulfonylurea, glibenclamide is also widely used in treating type 2 diabetes, which is a generic formulation, and its combination with metformin is an available therapy in the clinic. Binding of glibenclamide to K_ATP_ (SUR1-Kir6.2) enables cell depolarization, and in turn induces an influx of calcium to promote the secretion of insulin, mediating hypoglycemic action (Rorsman and Ashcroft, 2018; Glibenclamide: a review, 1971), a mechanism of action being identical to other sulfonylureas. The most frequent side effects of glibenclamide are hypoglycemia and weight gain (Gangji et al., 2007; Glibenclamide: a review, 1971). Although the principal cause of pharmacological actions of sulfonylureas is determined, considerable data have accrued suggesting that multiple targets, in addition to K_ATP_ channels, are impacted by these chemicals to induce pleiotropic effects, which may be possibly responsible for the pharmacological effectiveness and adverse effects. As an example, several sulfonylurea drugs, such as tolbutamide, directly serve to impinge the Epac2A/Rap1 signaling (Zhang et al., 2009), which is essential for the cAMP-induced insulin secretion in pancreatic β-cell (Shibasaki et al., 2007), suggestive of an additional functioning way in promoting the insulin release.

Besides K_ATP_ channels, other different types of ion channels, including K^+^ channels such as two-pore domain K^+^ (K_2P_) channels, calcium-activated K^+^ (K_Ca_^2+^) channels, voltage-gated K^+^ (Kv) channels, and inwardly rectifying K^+^ (Kir) channels, as well as voltage-gated Ca^2+^ channels (VGCCs), Na^+^ channels, transient receptor potential (TRP) channels and Cl^−^ channels are present in β-cells in the pancreatic islets of Langerhans (Thompson and Satin, 2021), with substantial functional roles in governing the resting membrane potential and tuning the action potential of target cells (Thompson and Satin, 2021; Islam, 2020). Some channels could subsequently regulate the Ca^2+^ entry for glucose-stimulated insulin secretion (GSIS) in β-cells. For example, TRP channels, including TRPC1, TRPM2, TRPM3, TRPM4, TRPM7, TRPP1, TRPML1, and TRPML3 channels, expressed in human β-cells at relatively high levels serve to enhance the intracellular Ca^2+^ levels by either direct ways, or indirectly facilitating membrane depolarization, making Ca^2+^ influx via voltage-gated Ca^2+^ channels (Islam, 2020).

The electrical activity of β-cells regulated by these ion channels is related to insulin release in response to glucose stimulation, especially for Kv2.1, which is crucial for shaping insulin secretion (Fu et al., 2017; Jacobson and Shyng, 2020). The delayed rectifier K^+^ currents largely carried by Kv2.1 channels, which are encoded by *KCNB1* and abundantly expressed in β-cells (Braun et al., 2008), are required for the repolarization of action potentials important to the firing frequency of β-cells, and a resultant elevation of glucose-stimulated insulin secretion (GSIS) is evident in Kv2.1 (−/−) mice (Li et al., 2013). Theoretically, both K_ATP_ channels and other K^+^ channels are able to exhibit similar effects, possibly with varying potency, in tuning the insulin secretion of pancreatic β-cells, due to these channels possessing similar regulatory actions in the electrical excitability. As such, the blockage of K^+^ channels but not K_ATP_ channels, including Kv, BK_Ca_, TASK-1 and SK4 could lead to an enhancement of insulin release in human islets (Henquin et al., 2017). As an example, a significant and additional increase of insulin secretion resulting from dampening Kv channels using blockers, such as 4-AP and TEA, is observed following inhibiting K_ATP_ channels with the application of tolbutamide (Su et al., 2001). The influence of other ion channels on modulating glycemic levels also is validated. For instance, the oscillatory change of cytosolic Ca^2+^ mediated by VGCCs after glucose stimulation, which is critical for insulin release, is declined in Trpm5 (−/−) pancreatic islets, making an impaired glucose tolerance (Colsoul et al., 2010). In human pancreatic beta-cells, exposure to tetrodotoxin (TTX) conducts a blocking action of Na^+^ currents and the resulting reduction of both transient and sustained insulin secretion (Barnett et al., 1995).

Compared to other ion channels, obviously, K_ATP_ channel is more essential in modulating the insulin secretion and glucose homeostasis, possibly due to linking cellular metabolism to electrical activity. Thus, an interesting question of whether other ion channels but not K_ATP_ channels could also be targeted by sulfonylureas is raised, the scenario of which may somehow contribute to the effectiveness and side effects of these drugs. Indeed, accumulating evidence uncovers that sulfonylureas are capable of impacting diverse ion channels, with the exception of K_ATP_ channels, in the direct and indirect ways, implying a complicate mechanism underlying antidiabetic actions of these chemicals and a novel way of glycemic control that differs from sulfonylureas inhibiting K_ATP_. In the context of present review, we display the related evidence of sulfonylureas affecting diverse ion channels, although the data being, to date, still limited, providing valuable clues to explain the clinical phenomena and to explore the underlying mechanism.

## 2 Direct impact of sulfonylureas on ion channels besides K_ATP_ channels

### 2.1 K^+^ channels

The repolarization of glucose-induced action potential (AP) in pancreatic β-cells is attributed to K^+^ efflux via several K^+^ channels such as Kv channels, the process of which governs the duration of AP and subsequent Ca^2+^ influx crucial for secreting insulin. The varying level expression of Kv channels, including Kv1.3, Kv1.4, Kv1.5, Kv1.6, Kv1.7, Kv2.1, Kv2.2 and Kv11.1 (Matsuda et al., 2018; MacDonald et al., 2001; Kalman et al., 1998; Segerstolpe et al., 2016), is observed in pancreatic β-cells, some mutations of which are closely associated with the enhanced risk of developing T2D (Kalman et al., 1998).

Available data, although limited, reveal that sulfonylureas directly serve to impact the K^+^ channels besides K_ATP_ channels, exerting an influence on the insulin secretion and adverse effects. In rat pancreatic β-cells, glyburide has an inhibitory effect on the Kv currents in a concentration-dependent manner, acting synergistically with its blockage of K_ATP_ channels to facilitate therapeutic efficacy (Hu and Wang, 2001).

The delayed-rectifier Kv1.3 channels serve as a potential modulator in immune response, due to its regulation in electrical activity of immune cells such as T lymphocytes (Grishkan et al., 2015). Reported data suggest that the ablation of Kv1.3 genes, which also is abundantly expressed in fat, liver and skeletal muscle (Pérez-Verdaguer et al., 2018; Tschritter et al., 2006), and channel inhibition lead to an enhanced peripheral insulin sensitivity via promoting the translocation of the glucose transporter, GLUT4, to the membrane surface (Xu et al., 2004), meaning Kv1.3 being as a pharmacologic target for glycemic control. Interestingly, the hypoglycemic drug glibenclamide also exhibits an inhibitory effect on Kv1.3 channels cloned from rabbit kidney (Yao et al., 1996); together this with the evidence in which Kv1.3 immunostaining is evident in the cytoplasm rather than membrane of pancreatic β-cells (Matsuda et al., 2018) indicate that Kv1.3 could be responsible, at least in part, for the effects of glibenclamide on insulin secretion and sensitivity.

Besides cardiomyocytes and neural cells, the hERG channel (Kv11.1) encoded by human *ether-a-go-go*-related gene, which is associated with long-QT syndrome type 2 (LQT2), also is expressed in pancreatic α and β cells, participating in the secretion of glucagon and insulin, respectively (Hardy et al., 2009), due to its substantial functional role in tuning the electric activity, such as the action potential repolarization. The clinical study reveals that the reduced blood glucose and elevated risk of hypoglycemia are present in LQT2 patients because of increased secretion of GLP-1 (glucagon-like peptide-1), GIP (glucose-dependent insulinotropic polypeptide) and insulin, as well as lowered glucagon secretion, which is resulting from loss-of-function mutations in hERG channels (Hyltén-Cavallius et al., 2017). Correspondingly, the oral antidiabetic agent glibenclamide also reversibly inhibits the hERG channel-mediated currents (Rosati et al., 1998), suggesting being responsible for its adverse iatrogenic effect of QT prolongation and related arrhythmias, or partial pharmacological effectiveness.

### 2.2 Na^+^ channels

Expression of voltage-gated Na^+^ channels (VGSCs) is available in human pancreatic β-cells, with predominant levels for Na_V_1.6 and the remainder being Na_V_1.7, Na_V_1.3 and Na_V_1.2, which appears to account for large TTX-sensitive Na^+^ currents and be responsible for the upstroke of the action potential (Barnett et al., 1995; Braun et al., 2008; Rorsman and Ashcroft, 2018). Accordingly, decreased action potential amplitude and glucose-stimulated insulin secretion in human β-cells is manifested when blocking VGSCs (Braun et al., 2008; Barnett et al., 1995).

Interestingly, human pancreatic glandule and pancreatic islet β-cells express the epithelial sodium channel alpha subunit (α-ENaC) (Zhang et al., 2024), which could form functional amiloride-sensitive and voltage-independent ENaC channels when combining with β and γ subunits (Noreng et al., 2018). Moreover, overexpression of α-ENaC allows for a significant reduction of insulin content and glucose-induced insulin release possibly through the IRE1α/XBP1 and PERK/CHOP ER stress pathways (Zhang et al., 2024). Treatment with sulfonylurea drug glibenclamide enables an activation of ENaC via the extracellular loop or transmembrane domains of α-subunit (Renauld and Chraibi, 2009; Schni et al., 2003). Apparently, enhanced activity of ENaC by glibenclamide could be associated with antidiabetic effects, exhibiting a paradoxical scenario to declined insulin release arising from overexpression of α-ENaC (Zhang et al., 2024).

### 2.3 TRP channels

Several types of transient receptor potential (TRP) channels, including TRPC1, TRPM2, TRPM3, TRPM4, TRPM7, TRPP1, TRPML1, and TRPML3, have been identified in human β-cells (Islam, 2020; Matos et al., 2022), which are nonselective cation channels and may contribute to the background inward current of β-cells (Rorsman and Ashcroft, 2018). Emerging evidence indicates that TRP channels have also been implicated as an essential player in β-cell electrophysiology. For example, TRPM5 could induce the intracellular Ca^2+^ oscillations in pancreatic islets, and accordingly is a positive regulator of glucose-induced insulin release (Colsoul et al., 2010). Furthermore, the release of Ca^2+^ from intracellular stores is mediated through TRPM2, which is activated by intracellular ADPR in a lysosomal compartment (Lange et al., 2009). The modulation of TRP channels on insulin secretion is clinic relevance with a manifestation of TRPM3 being downregulated in human β-cells from T2D patients (Segerstolpe et al., 2016). PKC-dependent TRPM4 and TRPM5 activation could contribute to glucagon-like peptide 1 (GLP-1) stimulating insulin secretion in β cells (Shigeto et al., 2015), an explanation for therapeutic action of this incretin hormone.

High levels of TRPA1 channels are detectable in rodent pancreatic β-cells, and exposure to agonists of TRPA1 results in Ca^2+^ influx and induce the basal insulin release, suggestive of promotion of insulin secretion via the depolarization arising from both activation of TRPA1 and inhibition of K_ATP_ channels (Cao et al., 2012). Interestingly, Babes et al. reveal that antidiabetic drug glibenclamide activates TRPA1 in heterologous cells and primary sensory neurons, these effects of which are considered as glibenclamide-related adverse effects in diabetic patients (Babes et al., 2013).

### 2.4 Cl^−^ channels

Accumulating evidence unveils that volume-regulated anion channels (VRAC or SWELL1), cystic fibrosis transmembrane conductance regulator (CFTR) and Ca^2+^-activated Cl^−^ channels (CaCCs) are involved in regulating the insulin secretion, possibly via Cl^−^ efflux-mediated membrane depolarization (Jacobson and Shyng, 2020; Thompson and Satin, 2021; Best et al., 2010). As such, many cystic fibrosis (CF) patients could develop cystic fibrosis-related diabetes (CFRD) exhibiting insulin insufficiency, a significant extra-pulmonary comorbidity associated with the CF transmembrane conductance regulator (CFTR) (Guo et al., 2014; Koivula et al., 2016).

Like K_ATP_ channels, CFTR Cl^−^ channel, a member of the adenosine triphosphate (ATP) binding cassette (ABC) superfamily, is regulated by intracellular ATP with a cAMP/PKA-dependent feature (Tsai et al., 2010; Gregory et al., 1990). Interestingly, hydrolysis of ATP is essential for opening and closing of CFTR channels, but ATP reduces the activity of K_ATP_ channels composed of Kir6 and SUR subunits, both of which share several properties in their sulfonylurea binding sites (Sheppard and Robinson, 1997). Two sulfonylurea drugs, glibenclamide and tolbutamide, exhibit an open channel blockage on CFTR channels (Sheppard and Robinson, 1997; Schultz et al., 1996; Venglarik et al., 1996), and glibenclamide binds within the CFTR channel pore (Gupta and Linsdell, 2002). In addition, non-sulfonylurea hypoglycaemic agents, such as meglitinide and mitiglinide, also enable a voltage-dependent inhibition in heterologously expressed CFTR channels (Cai et al., 1999). No further interpretation regarding the pharmacological impact of these sulfonylurea drugs inhibiting CFTR on diabetes is provided by above authors. It is, however, worth noting that inhibition of sulfonylurea agents on CFTR is generally supposed not to be beneficial for reducing glucose levels, due to opening of CFTR inducing membrane depolarization and resultant insulin release, which should be addressed by further studies.

## 3 Indirect modulation: the suppression of sulfonylurea-mediated activation of Epac2A on diverse ion channels

Adenosine 3,5,-monophosphate (cAMP), an important second messenger, has been well established as a key regulator for the insulin release during the glucose stimuli, which is mediated via PKA-dependent manner and PKA-independent manner (as known as Epac2A way) (Shibasaki et al., 2014). The Epac family of guanine nucleotide exchange factors (GEFs) consists of two isoforms, Epac1 and Epac2, which serve to activate Ras-like small GTPases (Rap1 and Rap2) via catalyzing the exchange of bound GDP for GTP (Bos, 2006; Kawasaki et al., 1998; de Rooij et al., 1998). Epac protein possesses carboxyl-terminal catalytic regions, which comprise CDC-25 homology GEF domain (CDC25-HD), Ras association (RA) domain, and Ras exchange motif (REM) domain, as well as the amino-terminal regulatory regions including cAMP-binding domain (cNBDs) and a disheveled, Egl-10, pleckstrin (DEP) domain (Bos, 2006). There exist three isoforms of Epac2 proteins including Epac2A, Epac2B and Epac2C, which are highly expressed in the nervous system and endocrine tissues (Kawasaki et al., 1998; de Rooij et al., 2000). When cells are impinged by the extracellular stimuli, such as neurotransmitters, fatty acids, and hormones (Amisten et al., 2013), activated adenylate cyclases (ACs) binding to G protein-coupled receptors (GPCRs) can mediate cAMP production. Subsequently, the binding of elevated cAMP to the cNBD of Epac2A allows for the interaction of small GTPases Rap1 and CDC25-HD in catalytic regions, making activation of Rap1 by catalyzing the exchange of GDP to GTP (Rehmann et al., 2008). Active EPAC2A/Rap1 signal could conduct various biological actions throughout multiple biological systems, such as the modulation of insulin release, memory and inflammation (Robichaux and Cheng, 2018).

Previous studies uncovered that many sulfonylureas such as tolbutamide and glibenclamide, but not gliclazide, directly enabled the activation of Epac2A/Rap1 signaling in pancreatic β-cells, indicating an additional way of promoting insulin secretion, with exception of directly inhibiting K_ATP_ channels (Zhang et al., 2009). Activation of Epac2A allows for recruiting insulin granules from the readily releasable pool (RRP) (Seino et al., 2011), and inducing IP3-meidated enhancement of intracellular Ca^2+^ (Leech et al., 2011), accordingly potentiating the insulin secretion.

Interestingly, several K^+^ channels are also targeted by Epac, showing that possibly there is an indirect regulatory approach in which the impact of sulfonylureas on K^+^ channels arises from direct activation of Epac by these chemicals. A report that increased insulin secretion by activating P2Y purinergic receptor is attributed to Kv channel inhibition via cAMP/Epac/Kv channel pathway manifests an inhibitory influence of Epac on Kv channels (Zhang et al., 2015a) highly expressed in pancreatic β-cells, which requires the phosphatidylinositide 3-kinase (PI3K) as a critical mediator (Zhang et al., 2015b). Furthermore, activation of Epac leads to the blockage of K_ATP_ channels (Kang et al., 2008), a primary regulators of β-cell electrical activity, displaying that possibly there exists an indirect modulating way for insulin secretion, e.g., the inhibition of sulfonylurea-mediated activation of Epac on K^+^ channels. On the other hand, elevated cAMP levels by forskolin, an activator of adenylyl cyclase, can render a blocking action on Kv channels and subsequent prolongation of action potential duration, generating a resultant increase of insulin secretion (Liu et al., 2017), and although cAMP/PKA-dependent pathway could be responsible for this event (He et al., 2010), the involvement of Epac, a downstream effector of cAMP, in Kv channel inhibition, cannot be excluded. Interestingly, enhanced activity of PKA serves to promote the surface expression of Kv2.1 channels in pancreatic β-cells (Wu et al., 2015), an opposite effect which also appears to be a long-term action offered by modulation of gene transcription. Notably, while there exists three distinct Epac2 isoforms, e.g., Epac2A, Epac2B and Epac2C, only Epac2A is presented in pancreatic islet and exhibits functional roles in β-cells (Shibasaki et al., 2014). These findings, plus the observation in which the very low level of Epac1 is detectable in pancreatic islets (Song et al., 2011), imply that Epac2A is predominant downstream effector when cAMP triggers the PKA-independent signaling in pancreatic β-cells.

As an important controller in the repolarizing process of the β-cell action potential, the large-conductance calcium-activated K^+^ (BK_Ca_) channels also participate in modulating the depolarization-induced insulin release, evidenced by BK_Ca_ blockade enhancing human β-cell AP amplitude and insulin secretion (Braun et al., 2008). Nevertheless, a study whereby Epac serves to activate BK_Ca_ (Roberts et al., 2013) seems to cause the reduction of β-cell AP amplitude and insulin secretion, conflicting that activation of Epac2A by sulfonylureas allows for the suppression of K^+^ channels that contributes to insulin release (Zhang et al., 2009). Therefore, the indirect modulation of sulfonylureas on K^+^ channels influencing the insulin secretion could be more complicate due to the inconsistency of experimental observations. It is clear that tuning the K^+^ channels via sulfonylurea-mediated activation of Epac2A is an additional way to exhibit the pharmacological effectives, which awaits further characterization.

## 4 The impact of sulfonylureas on diverse ion channels and clinical translation

Closure of K_ATP_ channels and resultant elevation of cytosolic Ca^2+^ lead to activation of insulin exocytosis, which is considered as the basic pharmacological mechanism of sulfonylureas (Inagaki et al., 1995). Therefore, the blood glucose control offered by sulfonylurea blockage of K_ATP_ channels in pancreatic β-cells is a target-directed therapeutic approach. As described in this paper, collected data indicate that other ion channels besides K_ATP_ channels are also impacted by sulfonylureas, which could be responsible for pharmacological effectiveness and side effects. Thus, theoretically, other ion channels could be taken as targets to develop a new therapeutic approach for glycemic control. The scenario of sulfonylureas affecting other ion channels besides K_ATP_ channels could provide valuable insights into clinical translation for the treatment of T2DM. To address this issue, future meticulous investigation is certainly required to clarify the action of sulfonylureas on other ion channels in more detail. On the other hand, it is well-documented that various ion channels are essential for cell proliferation, and play important roles in migration and/or invasion of tumor cells (Kunzelmann, 2005). Interestingly, the anti-tumor effect of sulfonylurea drug glibenclamide is evident, which can be mediated by inhibiting several ion channels, such as K_ATP_ and Kv channels (Núñez et al., 2013; Zhou et al., 2003). Therefore, these published data suggest that effects of sulfonylureas on multiple ion channels may provide innovative clues to explore translational medicine for various diseases including diabetes.

## 5 Summary

**FIGURE 1:**
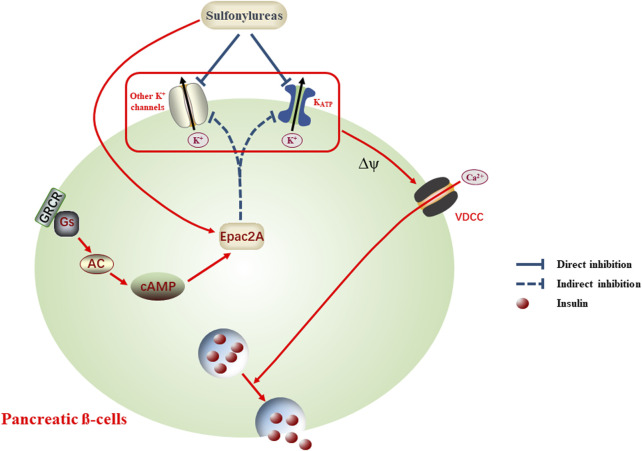
Schematic scenario of sulfonylureas impacting diverse K^+^ channels, an alternative explanation for the antidiabetic actions and adverse effects. Application of sulfonylureas conducts direct or indirect inhibition on diverse K^+^ channels, the latter being mediated by activating Epac2A. Dampening K^+^ channels generates the membrane depolarization (Δψ) and in turn open the voltage-dependent calcium channels (VDCC), allowing the influx of Ca^2+^ and accompanying insulin release, which may contribute to the reduced plasma glucose levels or adverse effects, such as hypoglycemia.

It is well known that sulfonylureas are successful clinical drugs to control the plasma glucose level in patients with T2DM for several decades. Although blocking the K_ATP_ channels by binding to the specific receptor of pancreatic β-cells is considered as a basic mechanism underlying the therapeutic effects, a growing body of evidence has suggested that these chemicals may also impact other targets, producing actions possibly responsible for pharmacological effectiveness and side effects. In this context, collected data are presented to manifest that sulfonylureas are capable of impacting other ion channels besides K_ATP_ channels, such as TRP and BK_Ca_, via direct and indirect ways, exhibiting the involvement of diverse ion channels in the modulation of insulin secretion and antidiabetic actions (see Table 1). It is hard to present a compatible schematic for all data, and a possible pattern of sulfonylureas impacting diverse K^+^ channels is shown in Figure 1. Of these data, contradictory observations mentioned above are evident, implying a complicated scenario and more measurements being needed in the future. Thus, additional experiments could be conducted to systematically explore the effects of sulfonylureas on other ion channels besides K_ATP_ channels, especially for some channels such as Kv2.1, which are closely linked to insulin release in β-cells (Fu et al., 2017; Jacobson and Shyng, 2020). It is worth noting that experimental design should be sophisticated and comprehensive, taking various influencing factors into account. For example, Mg^2+^-dependent inhibition of K_ATP_ by sulfonylureas, such as tolbutamide and glibenclamide, is evident, suggestive of the importance of Mg^2+^ in the impact of sulfonylureas on ion channels, and it may be factored into devising related experiments.

**TABLE 1:** Direct impact of sulfonylureas and indirect modulation of sulfonylureas targeting Epac on ion channels besides K_ATP_ channels.

Ion channels	Direct modulation by sulfonylureas	Indirect modulation of sulfonylureas targeting Epac
K^+^ channels	Inhibition of glibenclamide on Kv1.3 (Xu et al., 2004)	inhibitory influence of Epac on Kv channels (Kawasaki et al., 1998)
	Inhibition of glubiride on Kv in rat β-cells (Segerstolpe et al., 2016)	Activation of BK_Ca_ by Epac (Leech et al., 2011)
	Glibenclamide inhibition on hERG (Hyltén-Cavallius et al., 2017)	—
Na^+^ channels	Enhanced activity of ENaC by glibenclamide (Noreng et al., 2018; Renauld and Chraibi, 2009)	—
Cl^−^ channels	Blockage of glibenclamide and tolbutamide on CFTR channels (Gregory et al., 1990; Sheppard and Robinson, 1997; Schultz et al., 1996)	—
TRP channels	Activation of TRPA1 by glibenclamide (Cao et al., 2012)	—

Taken together, the findings of sulfonylureas impinging on several different ion channels expand the known pharmacological targets of these drugs, providing an alternative explanation for the antidiabetic actions and side effects.
